# Rock‐crushing derived hydrogen directly supports a methanogenic community: significance for the deep biosphere

**DOI:** 10.1111/1758-2229.12723

**Published:** 2018-12-26

**Authors:** Ronald John Parkes, Sabrina Berlendis, Erwan G. Roussel, Hasiliza Bahruji, Gordon Webster, Anthony Oldroyd, Andrew J. Weightman, Michael Bowker, Philip R. Davies, Henrik Sass

**Affiliations:** ^1^ School of Earth and Ocean Sciences Main Building, Park Place, Cardiff University Cardiff CF10 3AT Wales, UK; ^2^ Cardiff Catalysis Institute, School of Chemistry Cardiff University Cardiff, CF10 3AT Wales, UK; ^3^ School of Biosciences Sir Martin Evans Building, Cardiff University Museum Avenue Cardiff CF10 3AX Wales, UK

## Abstract

Microbial populations exist to great depths on Earth, but with apparently insufficient energy supply. Earthquake rock fracturing produces H_2_ from mechanochemical water splitting, however, microbial utilization of this widespread potential energy source has not been directly demonstrated. Here, we show experimentally that mechanochemically generated H_2_ from granite can be directly, long‐term, utilized by a CH_4_ producing microbial community. This is consistent with CH_4_ formation in subsurface rock fracturing in the environment. Our results not only support water splitting H_2_ generation as a potential deep biosphere energy source, but as an oxidant must also be produced, they suggest that there is also a respiratory oxidant supply in the subsurface which is independent of photosynthesis. This may explain the widespread distribution of facultative aerobes in subsurface environments. A range of common rocks were shown to produce mechanochemical H_2_, and hence, this process should be widespread in the subsurface, with the potential for considerable mineral fuelled CH_4_ production.

## Introduction

The majority of prokaryotes on Earth live in the subsurface and are present to depths in excess of 3 km (Parkes *et al*., 2014). These prokaryotes are far away from photosynthetically derived organic matter and oxygen and are under severe energy limitation (Hoehler and Jorgensen, 2013). Therefore, subsurface microorganisms maybe be more reliant on the geosphere for energy supply (Pedersen, 2000), including H_2_ which has a range of geosphere sources. For example: (i) oxidation of ferrous iron containing minerals, predominantly at elevated temperatures – serpentinization (Holm *et al*., 2015); (ii) radiolysis of water (Lin *et al*., 2005); (iii) pyrite formation from FeS and H_2_S (Drobner *et al*., 1990); and (iv) high temperature conversion of water in minerals into H_2_ and peroxy linkages (Freund, 1985). Low temperature (~20 °C) basalt weathering/oxidation had been suggested to fuel a H_2_‐based microbial ecosystem in the Columbia River Basalt Aquifer (Stevens and McKinley, 1995). However, this community subsequently was considered to be heterotrophic instead, as little H_2_ formation occurred under simulated *in situ* conditions and also because ferrous iron concentrations would have been limiting (Anderson *et al*., 1998). Despite this, total H_2_ flux in continental rocks has been suggested to be highly significant at 0.36–2.27 x 10^11^ mol per year (Lollar *et al*., 2014), and comparable to the seafloor hydrothermal H_2_ fluxes that support spectacular marine ecosystems. This flux would help explain the large terrestrial subsurface biosphere, but H_2_ from water radiolysis and serpentinization would be restricted to rocks with radioactive compounds or ferrous iron minerals respectively.

Another source of geologically‐generated H_2_ is from mechanochemical splitting of water due to free radical reactions on fractured rock surfaces (Kita *et al*., 1982; Freund *et al*., 2002) or rocks under tension (Balk *et al*., 2009). However, mechanochemical H_2_ formation is rarely considered as a deep biosphere energy source despite this process being widespread and not limited to a few specific rock types (Kita et al., 1982; Freund *et al*., 2002). Although fracturing is concentrated around earthquake zones (Wakita *et al*., 1980; Brauer *et al*., 2005), rock comminution during erosion (Telling *et al*., 2015) and seismic events (Sleep and Zoback, 2007), are also sources of mechanochemical H_2_ and together these should be widespread in the subsurface. Estimates of mechanochemically produced H_2_ at 3.4 × 10^16^ mol per year (Hirose *et al*., 2011; 2012) show that it is a larger global H_2_ source than serpentinization and water radiolysis combined. In addition, the presence of CH_4_ in earthquake zones (Brauer *et al*., 2005) suggests that some of this mechanically produced H_2_ is being used directly by subsurface methanogens.

However, it is unknown if the production rates and concentrations of mineral‐H_2_, the conditions for its production (e.g. temperature and pressures) and/or the by‐products of the reactions (e.g. highly reactive oxygen species), would actually enable utilization by anaerobic microbial communities. Investigating whether mechanically‐produced H_2_ can be directly utilized by prokaryotic communities is not only important for understanding deep biosphere energy sources, if a significant amount of this H_2_ is utilized to form CH_4_, this would also be important for accurate quantification of greenhouse gas formation and global warming. Furthermore, mechanochemical‐H_2_ formation may have been important for early life on Earth and could potentially maintain subsurface biospheres on other planets (McMahon *et al*., 2016). We, therefore, conducted laboratory rock‐crushing experiments under optimal conditions for H_2_‐utilizing methanogens to test whether mechanochemical‐H_2_ formation could directly fuel microbial activity, and hence, potentially microbial ecosystems.

## Results and discussion

### *Mineral‐H_2_ formation on crushing*

**Figure 1:**
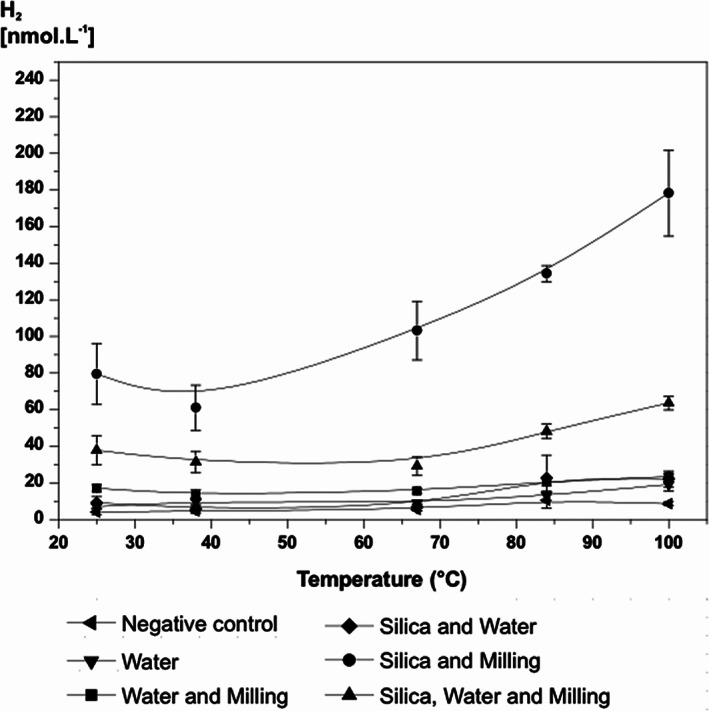
Effect of milling on H_2_ formation from silica between 25 and 100 °C (mean of triplicates and standard error bars shown).

To determine the mineral H_2_ formation conditions for subsequent microbial utilization, pure silica (2 g) in vials with aluminium balls under anaerobic conditions were heated at 25, 38, 67, 84 and 100 °C for 30 min and then contents ground using a ball mill (60 min, Supporting Information Fig. S1A; see Supporting Information for Experimental procedures). The vials were then heated for a further 30 min before headspace gas was analysed. Above ~40 °C H_2_ concentrations increased with temperature (*P* < 0.05), reaching 178 nmol H_2_ L^−1^ headspace at 100 °C for silica only with milling. All controls, including silica plus water, were not significantly different from an empty vial (Fig. 1). These results show that milling and silica were essential for producing significant H_2_, and that other potential sources of H_2_ on heating, such as thermal breakdown of organic matter contaminants and rubber stoppers, were negligible sources of H_2_ under the prevailing conditions. Furthermore, milling of water with silica produced considerably less H_2_ compared with silica without water (Fig. 1), suggesting that the added water reduced milling efficiency. This further emphasizes the importance of milling for H_2_ formation as does the experiment with silica plus water without milling which produced even less H_2_ (~20 nmol L^−1^). Dry grinding of minerals produces H_2_ with the water coming from between mineral grains or from reaction of hydroxyl groups (Kameda *et al*., 2004). Although H_2_ formation from silica, and granite, has been shown to increase with temperature, up to a maximum at ~200–220 °C (Kita *et al*., 1982), lower temperatures are required for direct coupling with microbial H_2_ utilization, as the upper temperature for prokaryotes and methanogenesis is around 120 °C (Takai *et al*., 2008). Hence, there is a compromise between the temperature required for maximum mechanochemical mineral‐H_2_ formation, and the temperature range enabling its direct microbial utilization. From the temperature range tested (Fig. 1) 67 °C was selected for further experiments to enable subsequent coupling with the deep‐sea, thermophilic methanogen *Methanothermococcus okinawensis* (growth optimum 60–65 °C, range 40–75 °C, Takai *et al*., 2002). Prokaryotes at similar thermophilic temperatures have been detected in deep, subsurface sediments (Roussel *et al*., 2008) and in water from deep rock fracture zones (Takai *et al*., 2003, Moser *et al*., 2005).

**Figure 2:**
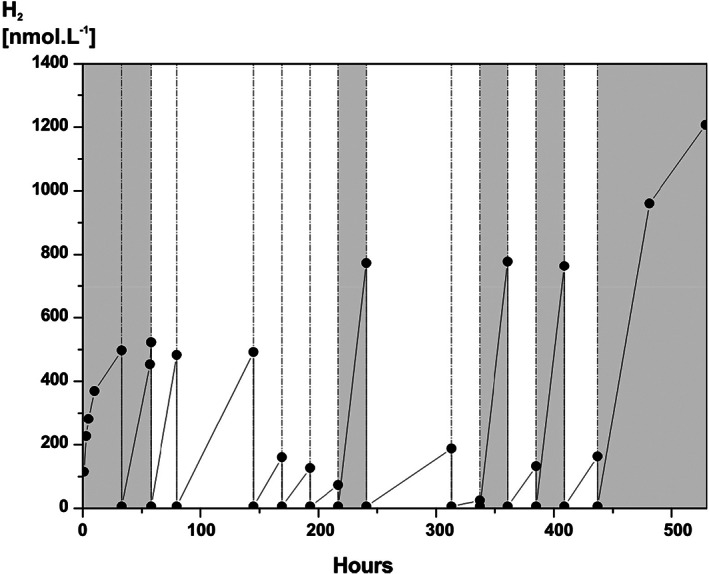
Effect of milling on H_2_ from silica at 67 °C. Dotted lines denote headspace flushing (x3 with oxygen free nitrogen). Shading shows milling intervals.

To enhance mineral‐H_2_ formation at 67 °C, silica milling was conducted in an oil bath (Supporting Information Fig. S1B) to provide extended periods of heated milling and this was combined with headspace flushing (Fig. 2). Initially with milling, there was rapid H_2_ formation decreasing slightly after ~30 h. However, after headspace flushing H_2_ rapidly returned to its original concentration, ~490 nmol L^−1^. Flushing was repeated another three times up to ~140 h, with the same result, even though milling had stopped after ~55 h. After a further three flushes up to 216 h, the amount of H_2_ produced reduced considerably (lowest ~90 nmol L^−1^), indicating that most reactive surfaces had been utilized. However, flushing had resulted in a ~2.5 times increase in the amount of H_2_ formed. Another period of milling increased H_2_ to above the initial concentration (~760 nmol L^−1^), although subsequent flushing resulted in only low H_2_ concentrations (Fig. 2). This sequence was repeated in another two milling periods, followed by an extra period without flushing which yielded the maximum H_2_ concentrations of 1213 nmol L^−1^, after a total of ~530 h. Free radical concentrations also increased with crushing time (Supporting Information Fig. S2) corresponding with increasing H_2_ formation. These results show that continuous H_2_ formation can be obtained by a mixture of (i) additional crushing, and (ii) H_2_ removal by headspace flushing. The latter is consistent with feedback inhibition and suggests that microbial H_2_ consumption might sustain or even enhance H_2_ formation. Cumulative H_2_ formation totalled 7186 nmol L^−1^.

Similar results were obtained with crushing basalt with total H_2_ production after ~120 h of 350 nmol L^−1^, with one initial milling period (~75 h). These results are similar to the initial H_2_ formation in low temperature basalt weathering experiments conducted previously by Anderson and colleagues (1998), who also suggested that initial H_2_ formation was possibly due to reactive mineral surfaces, however, our milling was probably more effective, resulting in significant H_2_ formation after headspace flushing, which did not occur in the Anderson *et al*. experiments. Milling at 67 °C for ~30 h also produced H_2_ from other minerals in the order of highest concentrations: granite > quartz > silica and borosilicate glass > basalt, ranging in maximum concentration from 1133 to 142 nmol L^−1^ (Supporting Information Fig. S3). Mineralogical changes with crushing granite, including reduction of quartz and formation of new minerals (Supporting Information Fig. S4, overall decrease in peaks labelled Q for quartz, including some also with other minerals and appearance of additional peaks respectively), confirmed that H_2_ generation occurred together with breakage of Si—O bonds in phyllosilicates, which together with free radical formation (Supporting Information Fig. S2) is consistent with mechanochemical reactions.

### *Coupling mineral derived H_2_ with methanogenesis*

For further experiments, the high H_2_ producing granite was used in increasing amounts (15–40 g) with 30 g giving maximum H_2_ production and then this amount was subsequently used as standard. Under these conditions ~500 μmol L^−1^ H_2_ was produced, but there was no H_2_ consumption or CH_4_ production when the system was inoculated with a *M. okinawensis* culture (Supporting Information Fig. S1D), despite repeated attempts (Supporting Information Fig. S5). Under our culture conditions the H_2_ threshold for significant CH_4_ production by *M. okinawensis* was between ~200 and 1500 μmol L^−1^, so sufficient H_2_ was present in our mineral experiments for the methanogen to use. However, mechanochemical splitting of water also produces highly reactive oxygen species (Balk *et al*., 2009), which could have inhibited this strictly, anaerobic methanogen. Subsurface environments are generally reducing (e.g. H_2_S and reduced metal species), so reactive oxygen species would be reduced, and/or be used directly or indirectly (oxidized products of reduced species ‐ thiosulfate and metal oxides) by facultative aerobes/anaerobes. This would not substantially occur in our pure culture methanogen experiments. Therefore, we specifically enriched a methanogenic community (see Supporting Information Fig. S6 and Experimental Procedures) under low oxygen concentrations (and low H_2,_ ~400 μmol L^−1^) to inoculate further experiments, which could both cope with oxidized species and produce CH_4_ from H_2_ (Fig. 4, Supporting Information Fig. S1). The same enrichment subculture was then used to inoculate all subsequent experiments (Supporting Information Fig. S6b), to ensure that the community composition was identical for each.

**Figure 3:**
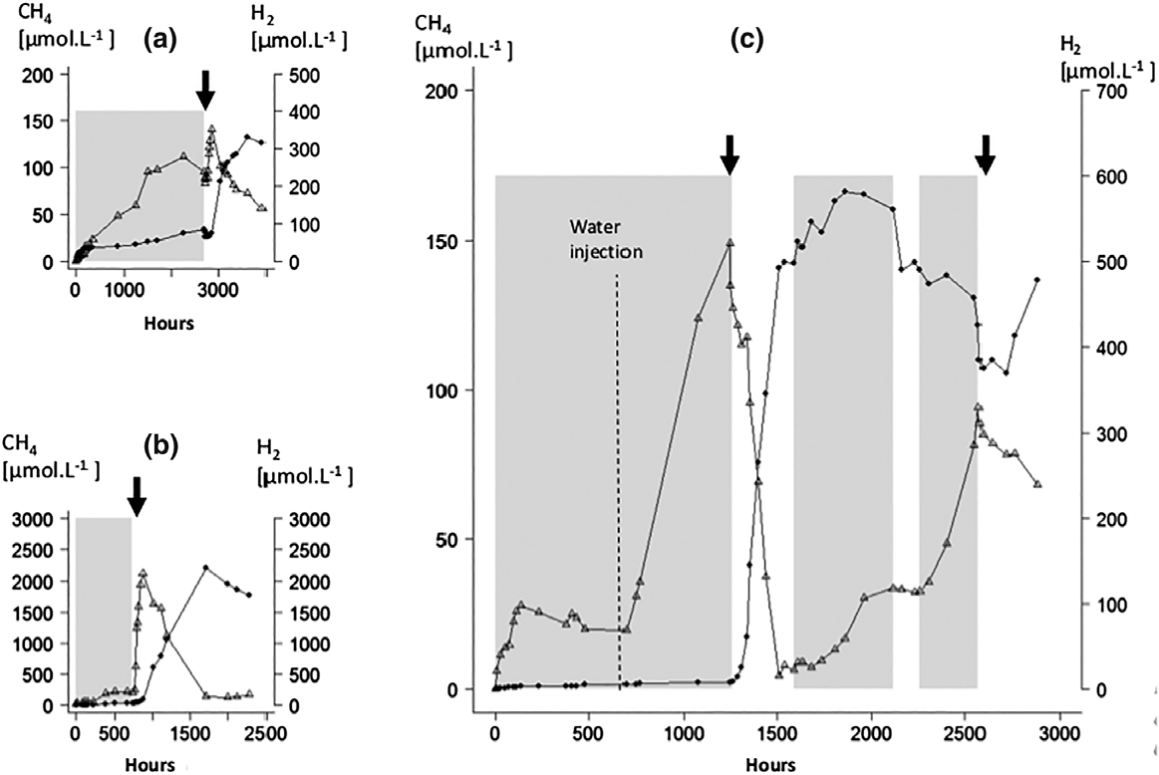
Granite milling experiments at 67 °C showing H_2_ consumption and CH_4_ production when inoculated with a methanogenic community.
Experiment 1: rotating with granite balls.Experiment 2 grinding with magnetic stirrer.Experiment 3: grinding with an abrasive resistant stirring bar. Triangle = H_2_, solid circle = CH_4_. Shading shows milling periods and arrow shows inoculation with methanogenic community.

Three experiments were conducted each with a different grinding mechanism to ensure that H_2_ formation was not restricted to a specific grinding process. Experiment 1 ‐ rotation with granite balls; Experiment 2 ‐ grinding with a magnetic stirring bar and Experiment 3 ‐ grinding with an abrasive resistant bar to prevent the iron magnet being exposed and contributing to H_2_ formation. All experiments resulted in H_2_ consumption and CH_4_ production after inoculation with the methanogenic community (Fig. 3). In Experiments 1 and 2, CH_4_ production was almost twice the amount expected from measured H_2_ consumption (4H_2_ + CO_2_ ➔ CH_4_ + 2H_2_O), which presumably reflects simultaneous mineral‐H_2_ production and H_2_ consumption by methanogens. In addition, enhanced H_2_ formation similar to the effect of headspace flushing previously documented, and of a similar magnitude (Fig. 2), may be occurring due to the methanogenic H_2_ consumption. In Experiment 3 (Fig. 3C), water (4 ml) was added after grinding for 682 h, to further increase H_2_ formation (~500 μmol L^−1^) and to demonstrate that the H_2_ increase previously observed on addition of the inoculum in Experiments 1 and 2 (Fig. 3A and B) was due to increased water availability after grinding. After ~1266 h the experiment was inoculated and almost immediately H_2_ was consumed and CH_4_ produced. By ~250 h incubation most of the H_2_ was removed (to ~20 μmol L^−1^) and CH_4_ then stabilized around 140 μmol L^−1^. Shortly after this, grinding was restarted (after 1587 h) and CH_4_ production restarted immediately, but for H_2_ there was a delay of ~140 h before concentrations increased, presumably due to its initial consumption for methanogenesis. During this second phase of grinding, CH_4_ and H_2_ concentrations plateaued at ~160 and 115 μmol L^−1^ respectively). Grinding was then stopped (2161 h) and H_2_ and CH_4_ (small decrease) production ceased. After ~60 h grinding was restarted and immediately H_2_ was produced, CH_4_ concentrations, however, decreased until the system was re‐inoculated (inoculum presumably dried out), after which H_2_ again was removed along with CH_4_ production, some 2800 h/120 days after the beginning of the experiment. The initial period of methanogenesis was much more rapid in this experiment compared with Experiments 1 and 2 (2–4 times, Fig. 3), and the H_2_:CH_4_ ratio was as expected for hydrogenotrophic methanogenesis. The second period of CH_4_ formation, however, was much slower, presumably reflecting the much lower H_2_ concentrations and the H_2_:CH_4_ ratio was similar to the Experiments 1 and 2. Probably the large and very rapid initial phase of H_2_ consumption in Experiment 3 masked the effect of continuing mineral H_2_ formation. In controls, including an inoculated empty crushing bottle, and an autoclaved inoculum, no coupled H_2_ removal and CH_4_ production occurred (Supporting Information Fig. S7). Some CH_4_ was released into the inoculated empty bottle control (max 26 μmol L^−1^), but this represented only a fraction of the CH_4_ produced in the inoculated mineral H_2_ experiments. The controls demonstrate that CH_4_ production was not a result of thermal breakdown of cells or organic matter in the inoculum. The rapid response to renewed mineral grinding (Fig. 3C) also demonstrates how tight and effective this mineral H_2_ methanogenesis system is.

### *The methanogenic community*

**Figure 4:**
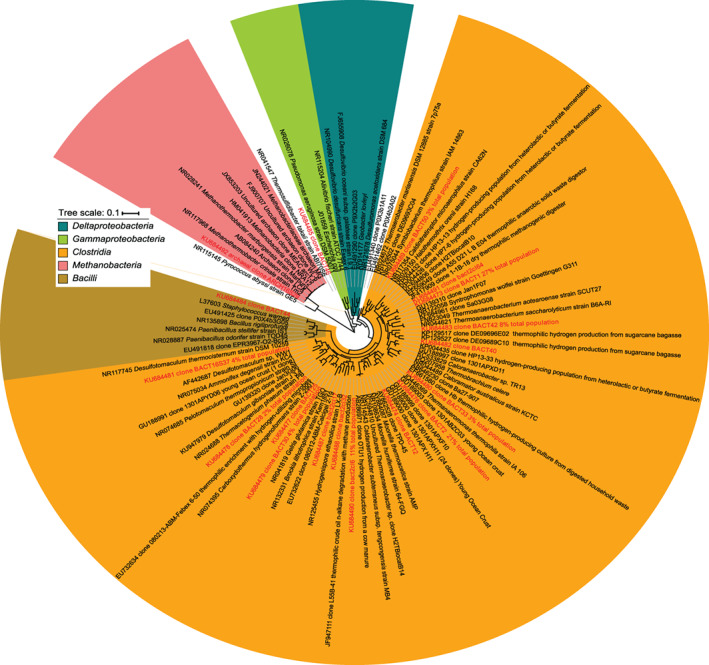
Phylogenetic tree of bacterial and archaeal 16S rRNA gene diversity in the methanogenic community inoculum. Neighbour‐joining tree prepared with MEGA 5.2.2 software (method: Jukes‐Cantor model, bootstrap test: 500 replicates) and edited with the Interactive Tree of Life (ITOL) using sequences aligned with the ClustalW2 program. Sequences detected in this study are highlighted in red and bold.

The composition of the methanogenic community was screened by methanogen functional *mcrA* gene and 16S rRNA gene sequence analysis (Supporting Information Fig. S8 and Fig. 4). Only one methanogenic archaeon was detected in the inoculum and this had 96% (*mcrA* gene) and 99% (16S rRNA gene) nucleotide sequence similarity to *Methanothermobacter crinale*, a methanogen often isolated from subsurface oil and gas reservoirs and thought to develop co‐operative relationships with *Bacteria* (Cheng *et al*., 2011). In addition to the methanogen, the methanogenic community also contained several bacterial 16S rRNA gene sequences (Fig. 4), predominantly thermophilic *Firmicutes*, belonging to the orders *Thermonanaerobacterales*, and *Clostridiales*, including *Desulfotomaculum* species, within the *Clostridia* class. Both of the above bacterial families commonly occur in the deep hot biosphere or deep subsurface environments (Aullo *et al*., 2013; Parkes *et al*., 2014, O'Sullivan *et al*., 2015, Purkamo *et al*., 2016). An association of methanogens with *Clostridiales* species have been shown to dominate in deep hot subterranean environments such as deep gold mines (Moser *et al*., 2003). Many bacterial sequences were related to cultured species (45%), including those from hot springs (Perevalova *et al*., 2013, *Brockia lithotrophica*, H_2_‐utilizing, obligate anaerobic, spore‐former), where a H_2_ driven methanogenic community has been documented (Chapelle *et al*., 2002); hot salty environments (Cayol *et al*., 1994, *Halothermothrix orenii*, an anaerobic, chemoorganotroph); and oil reservoirs (Nilsen *et al*., 1996), and some have known syntrophic interactions with methanogens (Nilsen *et al*., 1996, *Desulfotomaculum thermocisternum,* a thermophilic, H_2_‐utilizing, spore‐forming sulfate‐reducer). In addition, some *Desulfotomaculum* species have genes encoding for enzymes that can protect against reactive oxygen compounds (Spring *et al*., 2009). In our system, the presence of *Desulfotomaculum* species with the methanogen could help methanogenesis to occur despite the presence of oxidized compounds. One of the most common sequences (15%) was related to uncultured *Bacteria* colonizing young ocean crust (Fig. 4), which probably supports significant autotrophic microbial biomass (Bach and Edwards, 2003). Comparison of the bacteria in our methanogenic community (see Supporting Information Fig. S9) with those detected in H_2_‐utilizing SLIME environments (Stevens and McKinley, 1995) such as aging and young ocean crust (Cowen *et al*., 2003; Orcutt *et al*., 2011) or thermophilic methanogenic community fed from cathode‐derived hydrogen (Fu *et al*., 2013) shows that bacteria in our methanogenic community were representative of populations fuelled by H_2_.

### *Summary*

These results demonstrate considerable H_2_ formation from a range of common rocks and minerals by crushing induced mechanochemistry. This H_2_ formation can be sustained by repeated crushing under anaerobic conditions and at temperatures (25–100 °C) well within the range of prokaryotes. Higher temperatures produce even greater amounts of H_2_ (up to a maximum at ~200–220 °C, Kita *et al*., 1982) and this could diffuse upwards into the temperature limited base of the subsurface biosphere. Granite derived H_2_ was directly utilized by a CH_4_‐producing community in which the methanogen could effectively compete for H_2_ (over the 120 days of the experiment), and which was resilient to the oxidized species also mechanochemically produced, but not by a pure methanogen culture on its own. However, this production of oxidized species is also environmentally important as this could help sustain respiratory prokaryotes, independent of oxygen from surface photosynthesis, in the deep subsurface, including those using H_2_ or H_2_‐derived products, such as CH_4_. Subsurface oxidants may also help explain the considerable number of facultative aerobic prokaryotes in the deep subsurface (Pedersen, 1993; Miettinen *et al*., 2015).

Estimated earthquake‐derived H_2_ flux is five orders of magnitude higher (Hirose *et al*., 2012) than from radiolysis and serpentinization (Lollar *et al*., 2014) and, therefore, should be a much more important energy source for the deep biosphere, and provide a continuing fuel for deep CH_4_ formation and flux to the atmosphere, which is second only to wetland emissions (Etiope, 2012). If all of the estimated flux of mechanochemically derived H_2_ (Freund *et al*., 2002; Hirose *et al*., 2012) was converted to CH_4_ this would be ~1000 times higher than current estimates of geological CH_4_ greenhouse gas production (Kirschke *et al*., 2013). As tectonics on Earth probably occurred by ~3 Ga (Hirose *et al*., 2011), mechanochemical reaction products would also have been available to early life. Mechanochemistry from landslides, glacial bedrock comminution (Telling *et al*., 2015) and meteorite impacts add further to this tectonically driven water splitting, rock energy source, which could occur on many other planets (Hurowitz *et al*., 2007; Yin, 2012).

## Author contributions

RJP designed the project and wrote the paper. SB, EGR, GW and HB conducted the practical work, acquired and analysed data, and contributed to paper writing. All co‐authors contributed to the final version of the manuscript. Data supporting the paper and Experimental Procedures are presented in the Supplementary Information. GenBank Sequence accession numbers KU684473 to KU684492.

## Supporting information


**Fig. S1.** Apparatus used for milling experiments: A. Ball mill (150 r.p.m.). B. Rotary milling in a 67 °C oil‐bath with 25 ml Wheaton® vials (150 r.p.m.). C. Rotary milling in a 67 °C oil‐bath with 100 ml Duran® bottles. D. Grinding with a magnetic stirring bar in a beaker water‐bath on heated‐stirrer at 67 °C, with or without a separate methanogenic community inoculum.
**Fig. S2.** Free radical production from milled silica at 67 °C based on consumption of a radical scavenger (DPPH: Damm & Peukert (2009)). Black squares are milled silica; red circles are the non‐milled negative control.
**Fig. S3.** H_2_ and CO formation during milling a range of minerals at 67 °C. Circles = granite, triangles = quartz, squares = silica, star = borosilicate glass, diamonds = basalt.
**Fig. S4.** XRD profiles of fresh powdered granite initially used in the experiment (top) and the granite after crushing at 67 °C with a magnetic stirrer (bottom).
**Fig. S5.** Inoculation of granite derived H_2_ experiments with *Methanothermococcus okinawensis* at 67 °C and changes in CH_4_ (filled circle) and H_2_ concentrations (triangles). Shaded area represents the grinding period; dotted line denotes injection of sterile medium to enhance H_2_ production and arrows are injection of the methanogen pure culture. Replicate experiments a and b.
**Fig. S6.** Enrichment of air tolerant methanogenic community at 67 °C using sediments from the Tamar Estuary, UK in mineral medium: a) initial enrichment slurry with successive air additions, b) enrichment after successive subculture at low H_2_ concentrations in a vial mimicking experimental conditions. CH_4_ (filled circle) and H_2_ (triangles).
**Fig. S7.** Control experiments at 67 °C with a) autoclaved (x3) enrichment inoculated into an experiment with 30 g of crushed granite (shaded area is the grinding period) and H_2_ adjusted to ~300 μmol L^−1^. b) Active methanogenic enrichment inoculated into an empty device. Experiment 1 shown by solid lines and Experiment 2 by dashed lines. CH_4_ (filled circle) and H_2_ (triangles).
**Fig. S8.** Phylogenetic tree of methanogen functional *mcrA* gene clones from the methanogenic community inoculum. All clones were closely related (96%) to *Methanothermobacter crinale*.
**Fig. S9.** Comparison at the class‐level of bacterial 16S rRNA genes detected in this study with other studies of subsurface environments. 1: Parkes *et al*., (this study) crushing experiments, hot condition; 2: Fu *et al*. (2013) cathode hydrogen production sustaining methanogenic community, hot condition; 3: Cowen *et al*., (2003) aging ocean crust, hot condition; 4: Orcutt *et al*., (2011) young ocean crust, hot condition; 5: Diksma *et al*., (2016) dark C fixation in coastal marine sediments, cold condition; 6: Le Campion *et al*., unpublished, continental subsurface aquifer; 7–9: Dong *et al*., (2014) 1.8 km deep subsurface Cambrian sandstone reservoir, thermophilic; 10–12: Edlund *et al*., (2008) Baltic sea sediments, cold conditions (10 = redox depth ‐337 mV; 11 = redox depth − 169 mV; 12 = redox depth − 64 mV [b1]).
