# Supplementary material for: Rock‐crushing derived hydrogen directly supports a methanogenic community: significance for the deep biosphere
Source: Environ Microbiol Rep. 2018 Dec 26;11(2):165–72. doi: 10.1111/1758-2229.12723 (PMC7379504; doi:10.1111/1758-2229.12723)
Supplement: Supplementary file 1 — Fig. S1. Apparatus used for milling experiments: A. Ball mill (150 r.p.m.). B. Rotary milling in a 67 °C oil‐bath with 25 ml Wheaton® vials (150 r.p.m.). C. Rotary milling in a 67 °C oil‐bath with 100 ml Duran® bottles. D. Grinding with a magnetic stirring bar in a beaker water‐bath on heated‐stirrer at 67 °C, with or without a separate methanogenic community inoculum. Fig. S2. Free radical production from milled silica at 67 °C based on consumption of a radical scavenger (DPPH: Damm & Peukert (2009)). Black squares are milled silica; red circles are the non‐milled negative control. Fig. S3. H2 and CO formation during milling a range of minerals at 67 °C. Circles = granite, triangles = quartz, squares = silica, star = borosilicate glass, diamonds = basalt. Fig. S4. XRD profiles of fresh powdered granite initially used in the experiment (top) and the granite after crushing at 67 °C with a magnetic stirrer (bottom). Fig. S5. Inoculation of granite derived H2 experiments with Methanothermococcus okinawensis at 67 °C and changes in CH4 (filled circle) and H2 concentrations (triangles). Shaded area represents the grinding period; dotted line denotes injection of sterile medium to enhance H2 production and arrows are injection of the methanogen pure culture. Replicate experiments a and b. Fig. S6. Enrichment of air tolerant methanogenic community at 67 °C using sediments from the Tamar Estuary, UK in mineral medium: a) initial enrichment slurry with successive air additions, b) enrichment after successive subculture at low H2 concentrations in a vial mimicking experimental conditions. CH4 (filled circle) and H2 (triangles). Fig. S7. Control experiments at 67 °C with a) autoclaved (x3) enrichment inoculated into an experiment with 30 g of crushed granite (shaded area is the grinding period) and H2 adjusted to ~300 μmol L−1. b) Active methanogenic enrichment inoculated into an empty device. Experiment 1 shown by solid lines and Experiment 2 by dashed lines. CH4 (filled circ [file EMI4-11-165-s001.docx]

**Supporting Information**

**Experimental procedures**

**Mineral crushing and microbial inoculum experiments**

Minerals, including natural rock samples (granite and basalt) were obtained from Richard Tayler Minerals (Cobham, Surrey, UK, <http://richardtayler.co.uk>). All minerals were added to either crimp seal vials (25 ml, Fig S1, A), or larger Duran bottles with a 12 ml inner glass vial attached to the sealing stopper with nickel wires, into which the microbial inoculum was added (Fig S1, D) plus granite chunks (5-8 g), aluminium balls, or magnetic stirrer. The inoculum being in the inner vial enabled its direct exposure to the products of mineral crushing, without itself being crushed. Crushing (Fig. S1) was either with (A) ball mill, (B) rotation in a 67°C oil-bath (for long-term thermal stability and absence of evaporation, rpm ~150) or (C) and (D) via a magnetic stirrer with the bottle in a water-bath beaker on a stirring hot plate at 67°C (either with an abrasive resistant [300°C operating temperature] PEEK encapsulated SmCo 29 MGO magnet [V&P Scientific Inc., USA] or a standard laboratory stirring bar). All the systems were flushed with an anaerobic gas, normally N_2_/CO_2_ (80%/20%) or N_2_, and sealed. Glassware was routinely furnaced at 400°C for 180 mins, whilst the silica was furnaced at 1000°C for 30 mins to remove organic matter. Other natural minerals were not heated as this can increase H_2_ formation (Hurowitz *et al*., 2007). Only samples for headspace gas analysis were taken from experiments with a microbial inoculum to limit disturbance of the obligatory anaerobic methanogens, including a reduction in the already small culture volume and accidental introduction of air.

**Enrichment of a microaerophilic and thermophilic methanogenic culture**

Tamar estuary sediments were used for this enrichment as previously methanogenesis had been shown to occur around 67°C (Parkes *et al*., 2011), and some thermophilic bacteria related to deep biosphere communities were also present (O'Sullivan *et al*., 2015). The sediment slurry was prepared in a modified 2 litre Duran bottle enabling a continuous flow of N_2_/CO_2_ (80%/20%) gas during addition of autoclaved artificial seawater (in g/L, NaCl: 26.5, MgCl_2_.6H_2_O: 1.28, CaCl_2_.2H_2_O: 0.15, KCl: 0.5, NH_4_Cl: 0.25, KH_2_PO_4_: 0.2, Na_2_SO_4_: 1.42). After cooling under N_2_/CO_2_ (80%/20%), 30 ml of sterile deoxygenated NaHCO_3_ solution (1 M) was added and the medium adjusted to pH 7.6 with sodium hydroxide (1 M). Tamar Estuary, UK sediment (250 g from 5 cm depth) was added, mixed, flushed with H_2_/CO_2_ (80%/20%) plus 20 ml of sterile air to produce microaerophilic conditions (Fig. S6a). Incubation was at 67°C. After each headspace gas measurement a further 20 ml of air was injected to ensure maintenance of microaerophilic conditions. Subcultures with lower H_2_ concentrations were conducted when the slurry H_2_ concentration had been almost entirely consumed in order to select a methanogenic community adapted to lower H_2_ concentrations. This subculture headspace was subsequently flushed and replaced with even lower H_2_ concentrations (~400 µmol L^-1^). Subcultures were still methanogenically active under the lower H_2_ concentrations (Fig. S6b) and were flushed with N_2_/CO_2_ (80%/20%), to remove H_2_ and CH_4_, before being used to inoculate (8-12 ml) the mineral H_2_ experiments through the Duran bottle stopper into the inner vial (Fig. 3). H_2_ was added to the subculture headspace to maintain the enrichment in-between experiments.

**Gas Analysis**

Headspace H_2_, CO_2_, and CH_4_, were analysed by gas chromatography (Perkin Elmer Arnel Clarus 500 Natural gas analyser (NGA) with a thermal conductivity detector [TCD] and a flame ionisation detector [FID] with argon and helium as carrier gas, respectively, oven temperature 110°C and detectors at 150°C [TCD] and 250 °C [FID]). As the NGA H_2_ detection limit was >70 ppm, a more sensitive H_2_ analysis (~5 ppb) with a reducing compound photometer (RCP, Peak laboratories, Menlo park California) was also used. Above ~20 ppm H_2_ had to be diluted with N_2_ before RCP analysis.

**Composition of the microaerophilic methanogenic culture**

DNA extraction, 16S rRNA and methanogen functional *mcrA* gene analysis was conducted as described (Webster *et al*., 2006). Briefly, after DNA extraction using the FastDNA SPIN kit for Soil Clones (MP Biomedicals), DNA was amplified with bacterial primers 27F/1492R, archaeal primers 109F/958R and *mcrA* primers ME1/ME2. PCR products were then cloned into the pGEM-T Easy Vector System (Promega) according to the manufacturer`s instructions, with optimized insert:vector ratios and overnight ligation at 4°C. Libraries were screened by PCR with M13, 16S rRNA gene or *mcrA* gene primers. Clones with verified inserts were randomly selected for sequencing (Eurofins Genomics, Wolverhampton, UK) using the following primers: primer 27F for bacterial 16S rRNA genes, primer 109F for archaeal 16S rRNA genes and primer M13f for *mcrA* genes: 126 clones for bacterial 16S rRNA genes; 52 clones for archaeal 16S rRNA genes; 20 clones for *mcrA* genes. Sequences were aligned using the ClustalW2 program (Larkin *et al*., 2007) and trimmed in Bioedit. For the final phylogenetic trees representative reads from OTUs were used with MEGA 5.2.2 software (Tamura *et al*., 2013) and trees were obtained by neighbour –joining method with Jukes-Cantor algorithm. Bootstrap values (500) were calculated for all major nodes. Final version of the phylogenetic tree was edited with the online software “Interactive Tree of Life version 3.4.3” available at [www.itol.embl.de](http://www.itol.embl.de) (Ciccarelli *et al*., 2006; Letunic & Bork 2016).

Popset containing all sequences related to clone libraries from several subsurface environments or thermophilic enrichments were selected on NCBI. On a Linux Shell, with the bioinformatic pipeline QIIME (Caporaso *et al*., 2010), all sequences were then combined with the command add qiime labels.py in a single fasta file with valid QIIME fasta labels based upon specific sample IDs specified in a mapping file. Then, OTU picking, taxonomy assignment and OTU table reconstruction were done using the workflow ‘pick_de_novo_otus.py’. The biom-formatted OTU table was then processed with R statistical software with the package “biom” version 0.3.12 (McMurdie *et al*., 2014) and “qiimer” (Bittinger, 2015).

**XRD measurements**

Minerals were detected by X-Ray Powder Diffraction using a Philips PW1710 Automated Powder Diffractometer with X-Rays generated by Copper (CuKα) Radiation at 35kV 40 mA. The computer software was PW1877 APD version 3.6 and the identification software used was Match3! Software Version 3.3 (Gražulis et al., 2009) and QUALX version 2.1 for Windows (Altomare et al., 2015).with the following reference databases: COD Inorganics reference database or ICDD PDF-2 database.

**References**

Altomare A, Corriero N, Cuocci C, Falcicchio A, Moliterni A & Rizzi R (2015) QUALX2.0: A qualitative phase analysis software using the freely available database POW-COD. J Appl Crystallog 48: 598-603.

Bittinger K (2015) qiimer: Work with QIIME output files in R. Version 0.9.4. <https://cran.r-project.org/web/packages/qiimer/>

Caporaso JG, Kuczynski J, Stombaugh J, *et al*. (2010) QIIME allows analysis of high-throughput community sequencing data. Nature Methods 7: 335-336.

Ciccarelli FD, Doerks T, Von Mering C, Creevey C, Snel B & Bork P (2006) Toward Automatic Reconstruction of a Highly Resolved Tree of Life. Science 311 (5765): 1283–1287.

Gražulis S, Chateigner D, Downs RT, Yokochi AFT, Quirós M, Lutterotti L, Manakova E, Butkus J, Moeck P & Le Bail A (2009). Crystallography Open Database–an open-access collection of crystal structures. J Appl Crystallog 42: 726-729.

Hurowitz JA, Tosca NJ, McLennan SM & Schoonen MAA (2007) Production of hydrogen peroxide in Martian and lunar soils. Earth Planet Sci Lett 255: 41-52.

Larkin MA, Blackshields G, Brown NP, *et al*. (2007) Clustal W and clustal X version 2.0. Bioinformatics 23: 2947-2948.

Letunic I & Bork P (2007) Interactive Tree Of Life (iTOL): an online tool for phylogenetic tree display and annotation. Bioinformatics 23: 127-128.

McMurdie PJB-FT (2014) biom: An interface package (beta) for the BIOM file format. Version 0.3.12. <http://biom-format.org/>

O'Sullivan LA, Roussel EG, Weightman AJ, Webster G, Hubert C, Bell E, Head I, Sass H & Parkes RJ (2015) Survival of *Desulfotomaculum* spores from estuarine sediments after serial autoclaving and high-temperature exposure. ISME J 9: 922–933.

Parkes R, Linnane C, Webster G, Sass H, Weightman A, Hornibrook E & Horsfield B (2011) Prokaryotes stimulate mineral H2 formation for the deep biosphere and subsequent thermogenic activity. Geology 39: 219-222.

Tamura K, Stecher G, Peterson D, Filipski A & Kumar S (2013) MEGA6: Molecular Evolutionary Genetics Analysis Version 6.0. Mol Biol Evol 30: 2725-2729.

Webster G, Parkes RJ, Cragg BA, Newberry CJ, Weightman AJ & Fry JC (2006) Prokaryotic community composition and biogeochemical processes in deep subseafloor sediments from the Peru Margin. FEMS Microbiol Ecol 58: 65-85.


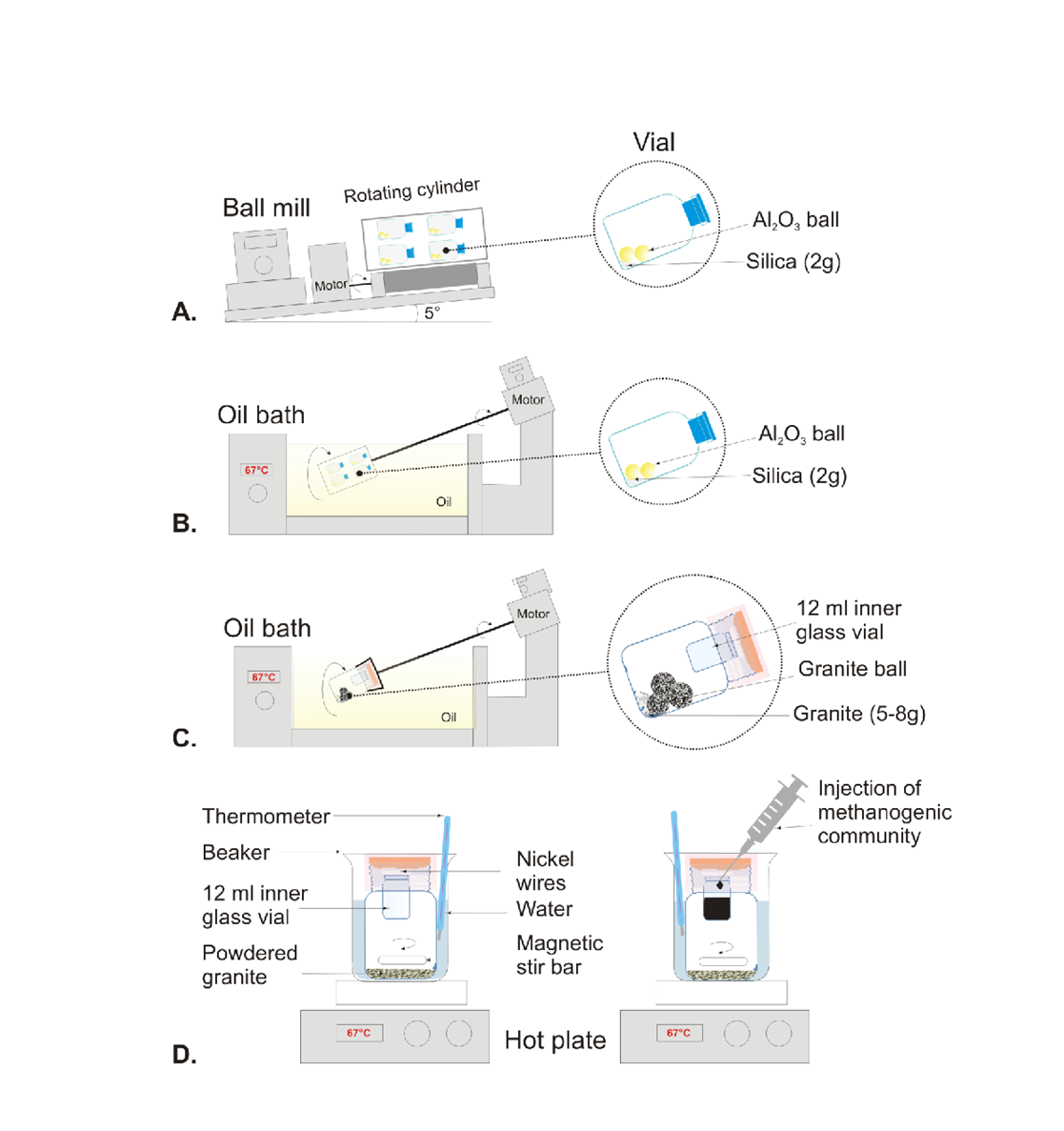


**Fig S1** Apparatus used for milling experiments: A. Ball mill (150 rpm). B. Rotary milling in a 67^o^C oil-bath with 25 ml Wheaton® vials (150 rpm). C. Rotary milling in a 67^o^C oil-bath with 100 ml Duran® bottles. D. Grinding with a magnetic stirring bar in a beaker water-bath on heated-stirrer at 67°C, with or without a separate methanogenic community inoculum.


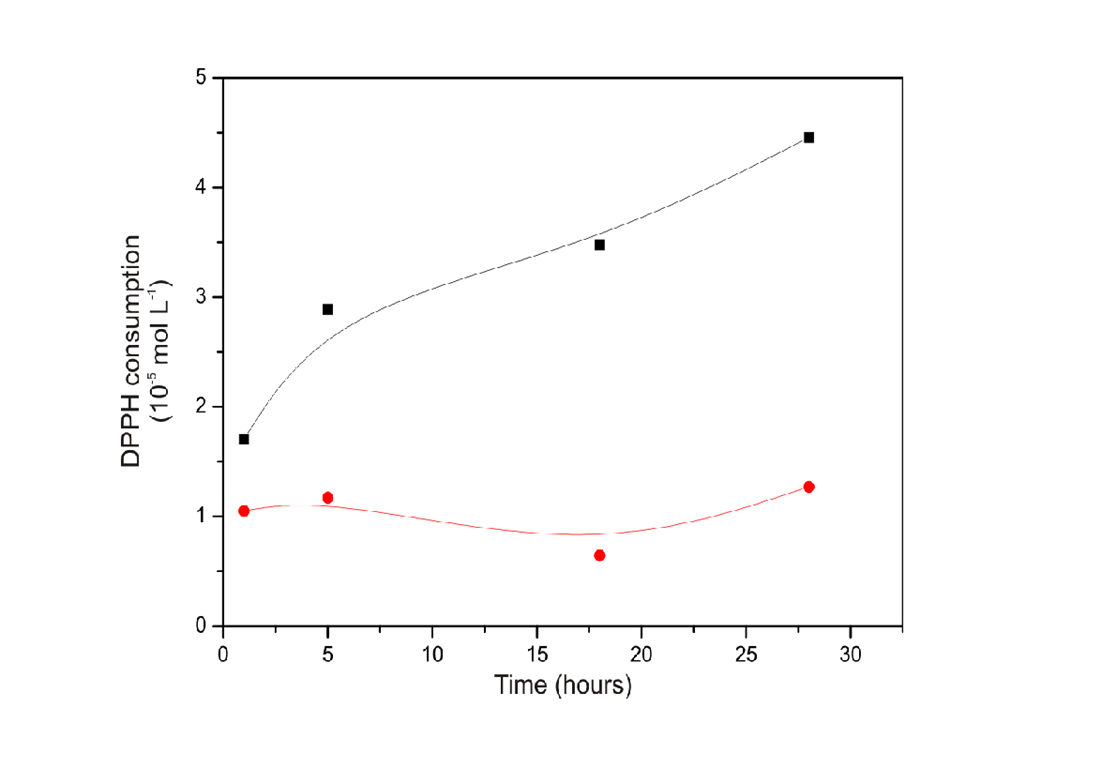


**Fig. S2** Free radical production from milled silica at 67°C based on consumption of a radical scavenger (DPPH: Damm & Peukert (2009)). Black squares are milled silica; red circles are the non-milled negative control.


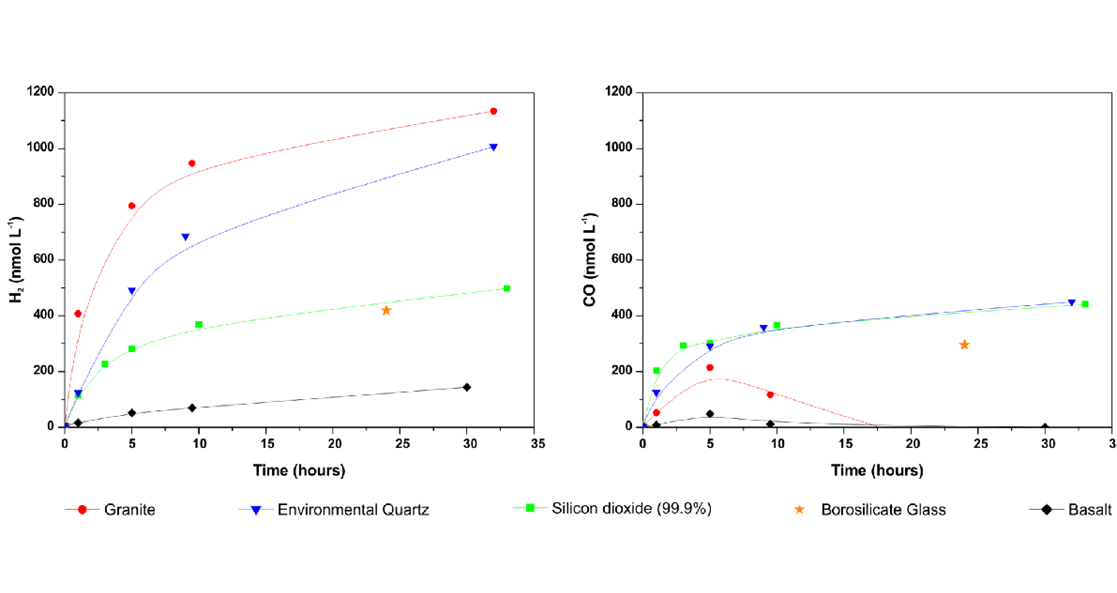


**Fig. S3** H_2_ and CO formation during milling a range of minerals at 67°C. Circles = granite, triangles = quartz, squares = silica, star = borosilicate glass, diamonds = basalt.


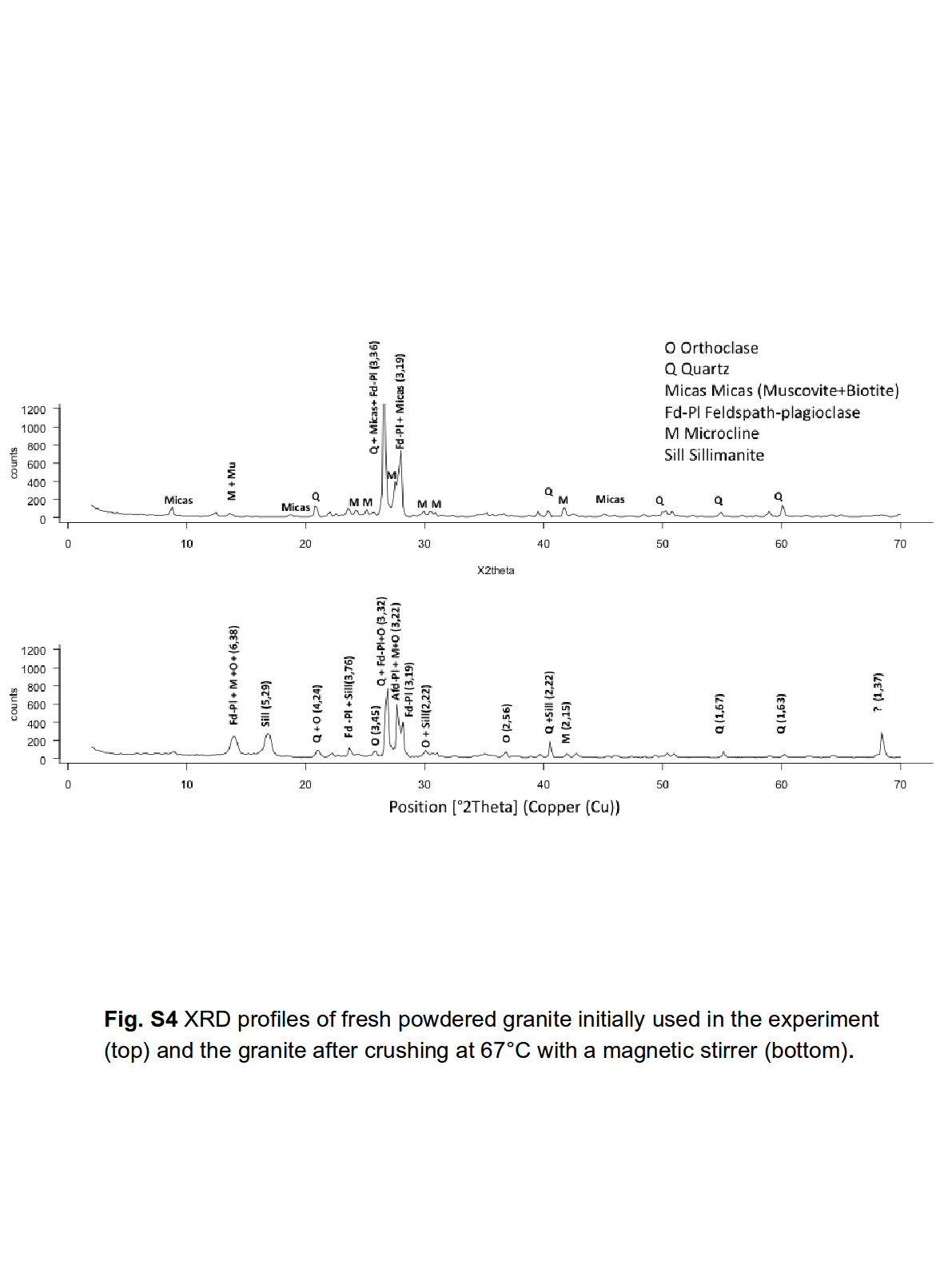


**Fig. S4** XRD profiles of fresh powdered granite initially used in the experiment (top) and the granite after crushing at 67°C with a magnetic stirrer (bottom).


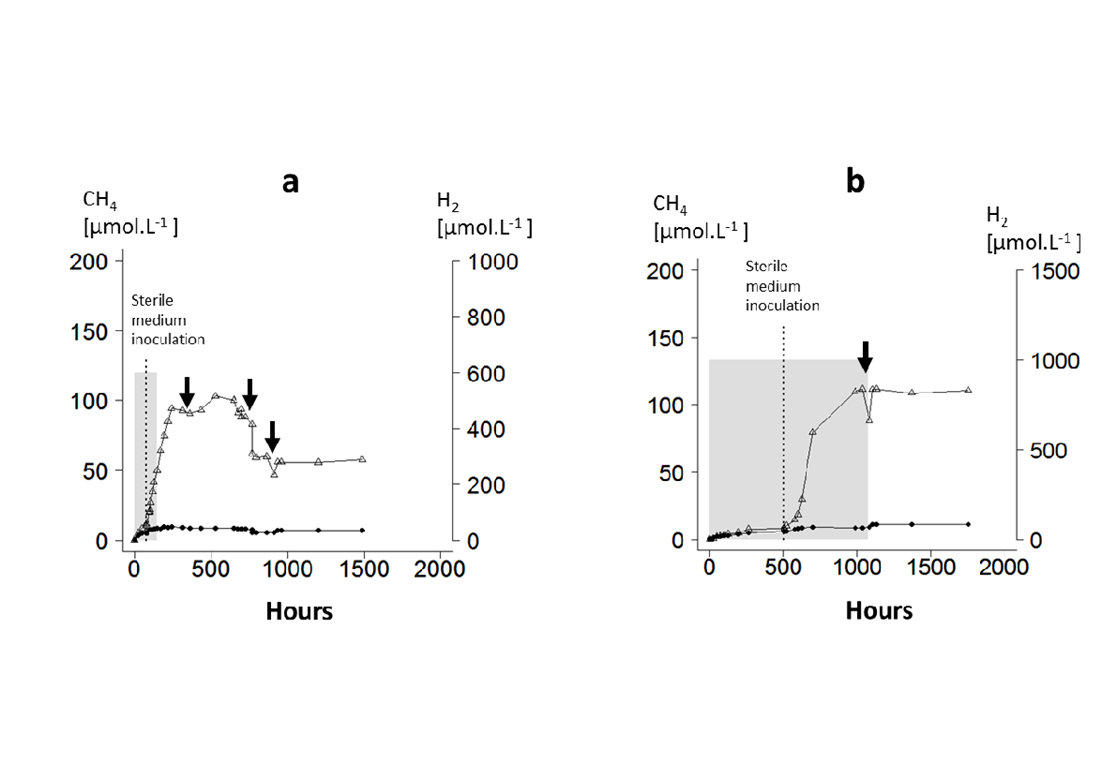


**Fig. S5** Inoculation of granite derived H_2_ experiments with *Methanothermococcus okinawensis* at 67°C and changes in CH_4_ (filled circle) and H_2_ concentrations (triangles). Shaded area represents the grinding period; dotted line denotes injection of sterile medium to enhance H_2_ production and arrows are injection of the methanogen pure culture. Replicate experiments a and b.


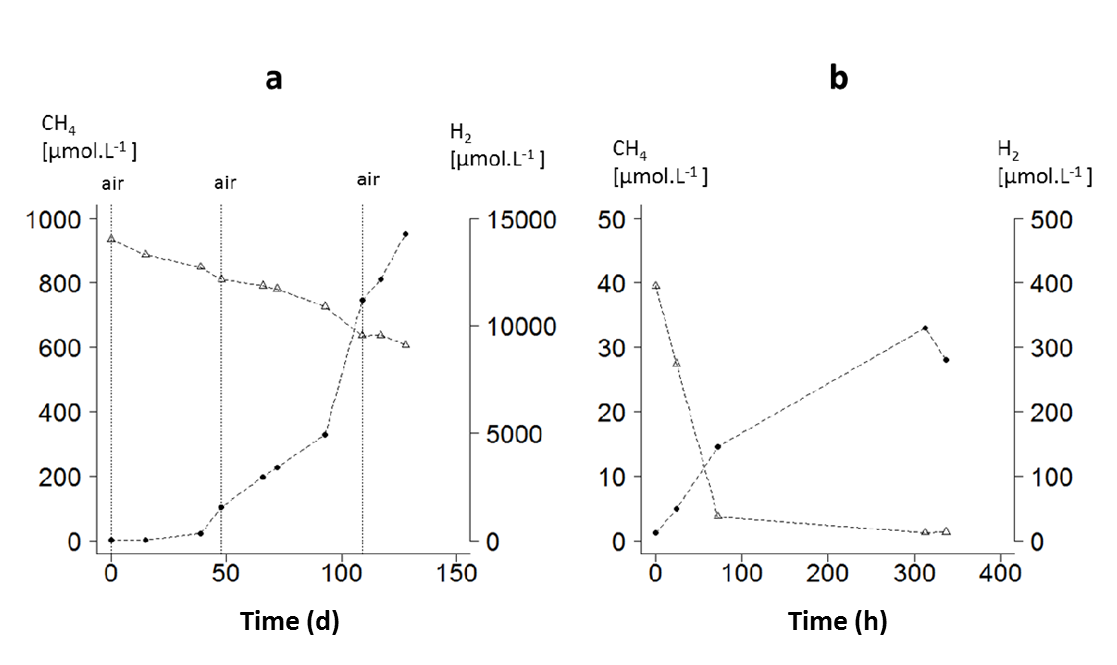


**Fig. S6** Enrichment of air tolerant methanogenic community at 67°C using sediments from the Tamar Estuary, UK in mineral medium: a) initial enrichment slurry with successive air additions, b) enrichment after successive subculture at low H_2_ concentrations in a vial mimicking experimental conditions. CH_4_ (filled circle) and H_2_ (triangles).

Fig. S7


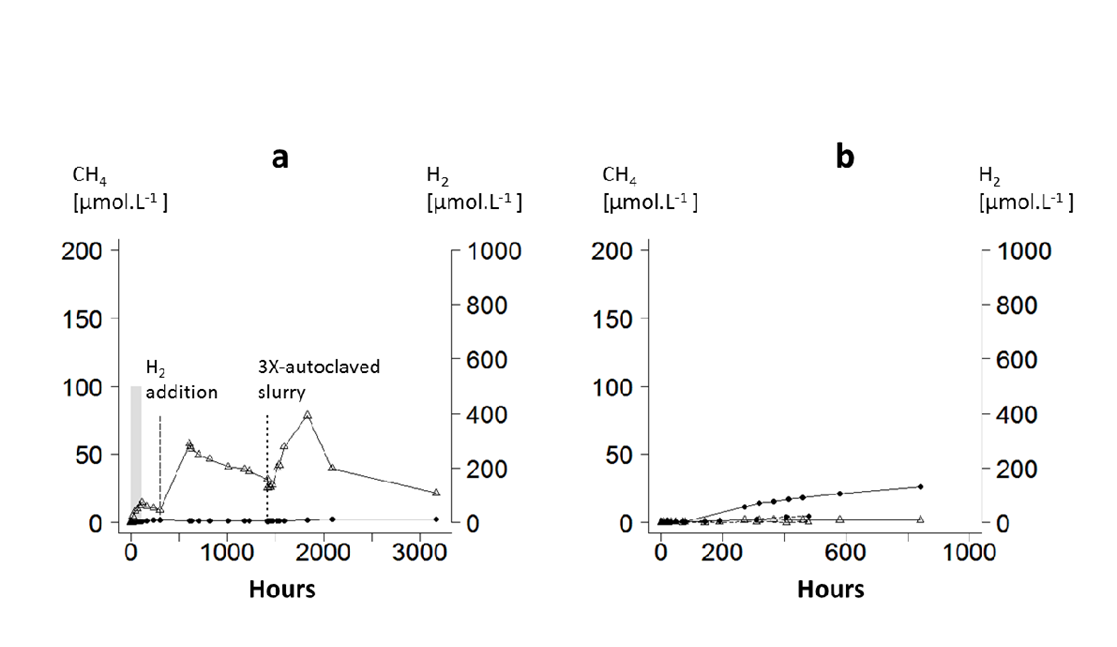


**Fig. S7** Control experiments at 67°C with a) autoclaved (x3) enrichment inoculated into an experiment with 30 g of crushed granite (shaded area is the grinding period) and H_2_ adjusted to ~300 µmol L^-1^. b) Active methanogenic enrichment inoculated into an empty device. Experiment 1 shown by solid lines and Experiment 2 by dashed lines. CH_4_ (filled circle) and H_2_ (triangles).


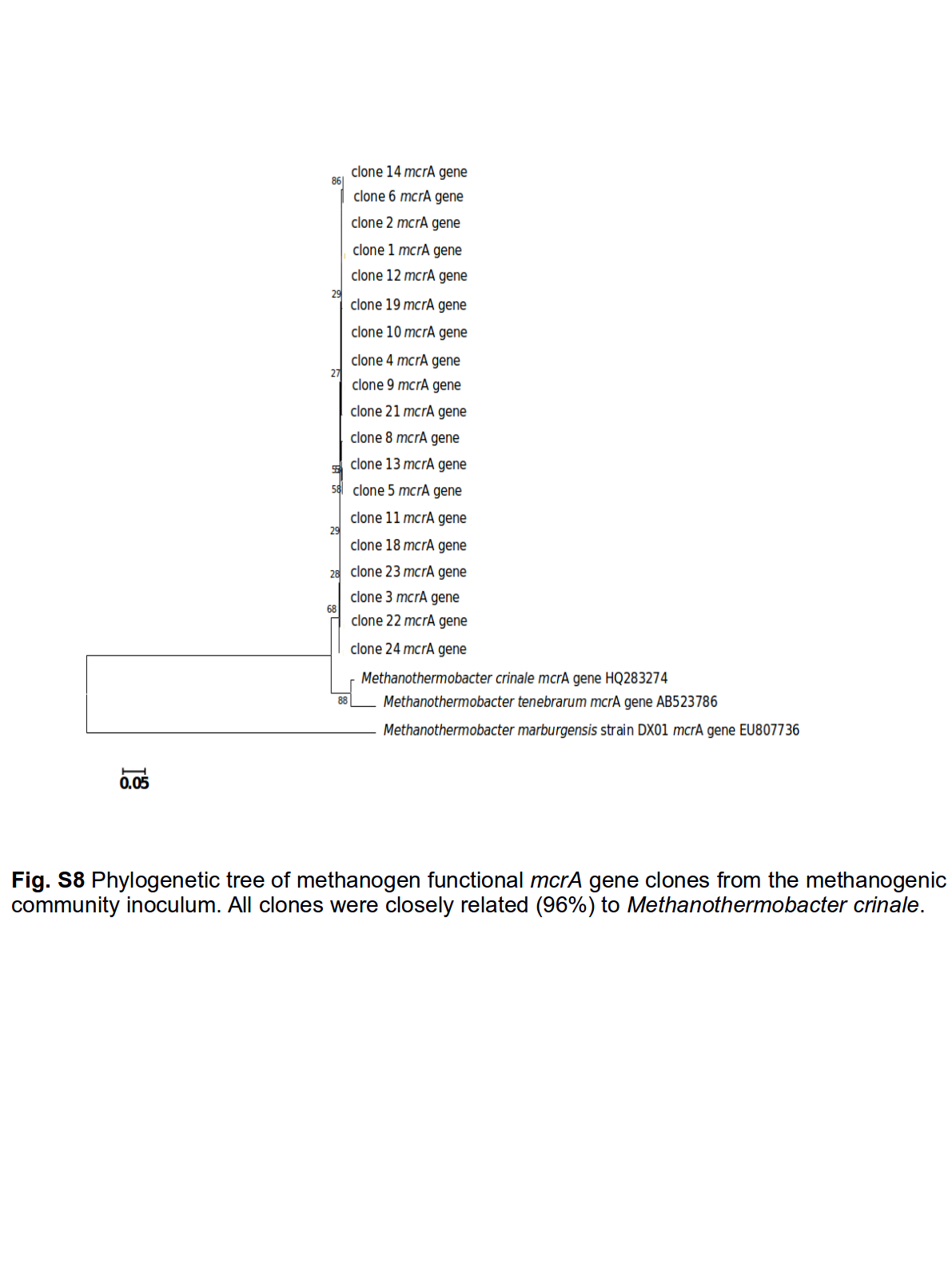
**Fig. S8** Phylogenetic tree of methanogen functional *mcrA* gene clones from the methanogenic community inoculum. All clones were closely related (96%) to *Methanothermobacter crinale*.


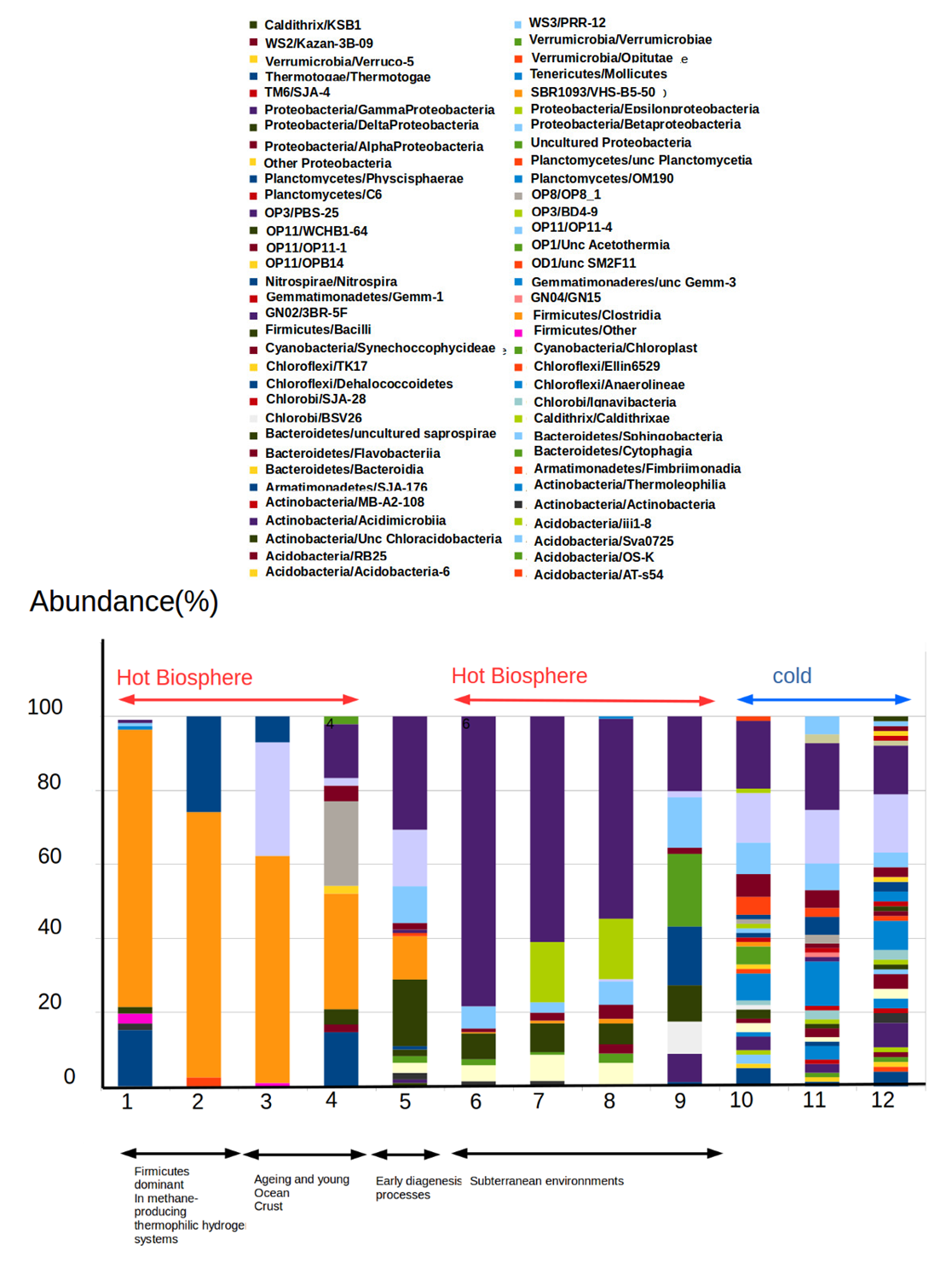
**Fig. S9** Comparison at the class-level of bacterial 16S rRNA genes detected in this study with other studies of subsurface environments. 1: Parkes *et al*., (this study) crushing experiments, hot condition; 2: Fu *et al*., (2013) cathode hydrogen production sustaining methanogenic community, hot condition; 3: Cowen *et al*., (2003) aging ocean crust, hot condition; 4: Orcutt *et al*., (2011) young ocean crust, hot condition; 5: Diksma *et al*., (2016) dark C fixation in coastal marine sediments, cold condition; 6: Le Campion *et al*., unpublished, continental subsurface aquifer; 7-9: Dong *et al*., (2014) 1.8 km deep subsurface Cambrian sandstone reservoir, thermophilic; 10-12: Edlund *et al*., (2008) Baltic sea sediments, cold conditions (10 = redox depth -337mV; 11 = redox depth -169 mV; 12 = redox depth -64 mV [b1]).

**Extra References (Figures S2 & S9)**

Cowen JP, Giovannoni SJ, Kenig F, Johnson HP, Butterfield D, Rappe MS, Hutnak M & Lam P (2003) Fluids from aging-ocean crust that support microbial life. Science 299: 120-123.

Damm C, & Peukert W (2009) Kinetics of radical formation during the mechanical activation of quartz. Langmuir 25: 2264-2270.

Diksma S, Bischof K, Fuchs BM, *et al*. (2016) Ubiquitous Gammaproteobacteria dominate dark carbon fixation in coastal sediments. ISME Journal 10: 1939-1953.

Dong Y, Kumar CG, Chia N, *et al*. (2014) Halomonas sulfidaeris-dominated microbial community inhabits a 1.8 km-deep subsurface Cambrian Sandstone reservoir. Environmental Microbiology 16: 1695-1708.

Edlund A, Hårdeman F, Jansson JK, & Sjöling S (2008) Active bacterial community structure along vertical redox gradients in Baltic Sea sediment. Environmental Microbiology 10: 2051-2063.

Fu Q, Kobayashi H, Kuramochi Y, Xu J, Wakayama T, Maeda H & Sato K (2013) Bioelectrochemical analyses of a thermophilic biocathode catalyzing sustainable hydrogen production. Int J Hydrogen Energy 38: 15638-15645.

Orcutt BN, Bach W, Becker K, Fisher AT, Hentscher M, Toner BM, Wheat CG & Edwards KJ (2011) Colonization of subsurface microbial observatories deployed in young ocean crust. ISME J 5: 692-703.
